# Thymic stromal lymphopoietin-activated basophil promotes lung inflammation in mouse atopic march model

**DOI:** 10.3389/fimmu.2025.1573130

**Published:** 2025-05-15

**Authors:** Xu Li, Zizhuo Li, Mindan Tang, Kaoyuan Zhang, Ting Yang, Weilong Zhong, Bo Yu, Fang Wang, Xia Dou

**Affiliations:** ^1^ Department of Dermatology, Peking University Shenzhen Hospital, Shenzhen, China; ^2^ Institute of Dermatology, Shenzhen Peking University-the Hong Kong University of Science and Technology Medical Center, Shenzhen, China; ^3^ Shenzhen Key Laboratory for Translational Medicine of Dermatology, Biomedical Research Institute, Shenzhen Peking University-the Hong Kong University of Science and Technology Medical Center, Shenzhen, China; ^4^ Department of Dermatology, Dermatology Hospital of Southern Medical University, Guangzhou, China

**Keywords:** atopic dermatitis, atopic march, basophil, eosinophilic lung inflammation, thymic stromal lymphopoietin

## Abstract

**Background:**

Atopic dermatitis (AD), a prevalent inflammatory skin disease affecting 10%-20% of the population, is linked to the development of asthma through atopic march (AM). This study aims to explore the role of basophils in OVA-induced lung inflammation in the presence of AD-like skin lesions and investigate the potential contribution of thymic stromal lymphopoietin (TSLP) in activating basophils.

**Methods:**

Mouse AM models were established in C57BL/6 mice using MC903 and OVA epicutaneous sensitization, followed by intranasal OVA challenges. An intraperitoneal OVA-sensitized asthma model was employed as the control group. RNA-Seq analysis was conducted on lung CD45^+^ immune cells from these models. Histologic examinations, flow cytometry, and ELISA were used to examine the lung and systemic inflammatory response. Basophil depletion was achieved through intraperitoneal administration of anti-FcϵRIα mAb. The role of TSLP was investigated using TSLPR knockout mice.

**Results:**

As in the intraperitoneal sensitization model, AM model also induced eosinophilic lung inflammation in mice, resembling the AM process. The RNA-Seq analysis revealed differential gene expression, with genes related to basophils being prominent in AM model. Increased basophil activation and IL-4 production were observed in OVA epicutaneously sensitized mice. Basophil depletion attenuated the eosinophilic lung inflammation. TSLP levels increased with topical MC903, and TSLPR knockout reduced lung inflammation, suggesting TSLP is involved in basophil activation.

**Conclusion:**

Basophils play a crucial role in OVA-induced lung inflammation in the context of AD-like skin lesions, and TSLP appears to drive basophil activation. Understanding these interactions provides insights for potential therapeutic interventions in AM-associated conditions.

## 1 Introduction

Atopic dermatitis (AD) is one of the most common inflammatory skin diseases, affecting approximately 10%-20% of the global population (1, 2). It is characterized by intense itching and extensive eczema-like lesions, which significantly compromise patients’ quality of life and impose a heavy disease burden on their families and society (3, 4). The skin condition of AD usually begins in early childhood, around 3–6 months of age. As patients age, they may develop other atopic conditions such as asthma, allergic rhinitis, and food allergies, which is known as the atopic march (AM) (5, 6). Although the causality of the combined diseases is still controversial, the risk of other atopic conditions is elevated in children with AD (7) and adult patients with poorly controlled AD (8). Additionally, the risk of atopic comorbidity decreases significantly with the effective control of AD skin conditions (8).

The pathogenesis of AD involves interactions between the skin barrier dysfunction, microbiome, and type-2 immune response (9, 10). Skin barrier damage leads to the release of epithelial-derived cytokines, including thymic stromal lymphopoietin (TSLP). TSLP can activate inflammatory dendritic cells, which subsequently drive the polarization of Th2 cells, leading to the release of IL-4 and IL-13 (11). These cytokines contribute to the disruption of the epidermal barrier (12–14). A compromised skin barrier facilitates transcutaneous antigen sensitization, resulting in the generation of antigen-specific immunoglobulin E (IgE) (15). Upon re-exposure of other epithelial tissues to the same antigens, this process initiates an allergic inflammatory response (16). In murine models with skin subjected to shaving and tape-stripping, topical sensitization with ovalbumin (OVA), followed by intranasal antigen challenge, reliably establishes a model of OVA-induced allergic asthma. This model is characterized by hallmark features of airway inflammation, mucus hypersecretion, and airway hyperresponsiveness (17).

Basophils, a subset of myeloid cells characterized by their basophilic cytoplasmic granules, constitute less than 1% of peripheral blood leukocytes in humans and mice under normal physiological conditions (18, 19). These cells express the high-affinity IgE receptor FcϵRIα and, upon activation, release histamine, proteases, and Th2 cytokines such as IL-4 and IL-13 (20). Notably, basophil-derived IL-4 plays a pivotal role in promoting Th2 cell differentiation (21). Basophil activation can occur via IgE-dependent or IgE-independent pathways. In eosinophilic esophagitis (EoE), TSLP has been implicated in disease pathogenesis by activating basophils through IgE-independent mechanisms (22, 23). Moreover, IL-33 also interacts with basophils in EoE in an IgE-independent manner (24). In a murine model of OVA-induced food allergy, TSLP-mediated basophil activation led to significant IL-4 production by basophils, driving an inflammatory response (25). These findings underscore the role of TSLP in contributing to the development of atopic conditions through basophil activation.

This study aims to investigate the role of basophils in inducing lung inflammation through intranasal sensitization by OVA, in the presence of AD-like skin lesions in mice, and to explore the potential role of TSLP in activating basophils.

## 2 Materials and methods

### 2.1 Mice

C57BL/6 mice and *Tslpr^-/-^* (*Crlf2^-/-^*) mice were purchased from GemPharmatech Corporation Limited (Nanjing, China) and maintained in a specific-pathogen-free facility at Shenzhen Peking University-Hongkong University of Science and Technology Medical Center. Mice were used for experiments between 6 and 8 weeks of age. The animal experiments were approved by the Ethics Committee of Peking University Shenzhen Hospital.

### 2.2 OVA sensitization and intranasal challenge

For the OVA epicutaneous sensitization model (AM model), from Day 0 to Day 13, mice were topically treated daily with 4 nmol of calcipotriol (MC903) dissolved in ethanol, applied to both sides of the mouse ears, and concurrently administered 200 μg of OVA in 50 μL saline (4 mg/mL). To induce lung inflammation, mice were intranasally (i.n.) administered with 50 μg OVA in 25 μL saline (2 mg/mL) on 4 consecutive days (Day 14 to Day 17). The vehicle control group was treated with ethanol and saline. Mice were sacrificed for analysis 1 day after the last challenge. For the OVA intraperitoneal (i.p.) sensitization model (atopic asthma, AA model), 20 µg OVA and 2 mg aluminum dissolved in 200 µL saline were i.p. injected to mice once a week for 3 weeks. To induce lung inflammation, mice were i.n. challenged with 25 μl OVA on 4 consecutive days after sensitization. For both groups, serum, bronchial alveolar lavage fluid (BALF), lung, spleen, and skin tissue were collected 1 day after the last intranasal administration. Lung tissues were scissored on ice and digested with 1mg/mL Collagenase D for 45 minutes at 37°C to obtain a single cell suspension, which was then separated from 30% Percoll gradient (Cytiva) and filtrated to culture in RPMI 1640 medium with 0.6mg/mL OVA or 1µg/mL TSLP (Sino Biological) for 5 hours at 37°C. Two hours after the stimulation of OVA and TSLP, a protein transport inhibitor cocktail (eBioscience) was added. The cells were then harvested for flow cytometry analyses.

### 2.3 Histologic analysis

Portions of the lung tissue were fixed in 10% neutral formalin, embedded in paraffin, and sectioned. Lung sections were stained with hematoxylin and eosin (HE) and Periodic acid–Schiff (PAS). BALF was centrifuged on slides using the Shandon Cytospin system (ELITechGroup Benelux, Belgium), followed by Wright-Giemsa staining and classified cell counting.

### 2.4 RNA extraction and quantitative PCR analysis

The harvested tissues were excised and homogenized in Trizol Reagent (Invitrogen, Carlsbad, Calif, USA). Total RNA was extracted and cDNA was synthesized from total RNA using the PrimeScript™ RT reagent Kit (TaKaRa, Japan). Real-time quantitative PCR was performed using the Taqman method on a Bio-Rad CFX96 Real-Time System (Bio-RAD, Hercules, Calif, USA). The mRNA levels of the target gene were normalized to the gene expression levels of glyceraldehyde-3-phosphate dehydrogenase (GAPDH) and are shown as relative expression levels to the control group. The primer sequences are provided in **Supplementary Table S1**.

### 2.5 RNA-sequencing gene expression analysis

CD45^+^ cells were collected by CD45 MicroBeads (Miltenyi Biotec) and Dead Cell Removal Kit (Miltenyi Biotec) according to the manufacturer’s protocol. RNA-seq was screened and analyzed by the BGI Genomics DNBSEQ-G400 platform (Shenzhen, China). Essentially, differential expression analysis was performed using the DESeq2 (v1.4.5) with Q value ≤ 0.05 and |log2FC|≥ 1. Volcano plot, UpSet plot and Venn plot were performed on R software (version 4.2.1). Enrichment analyses were separately performed for DEGs by referring to the GO and KEGG databases by using the clusterProfiler package. An adjusted *p* value of < 0.05 was considered statistically significant. Additionally, STRING [[https://cn.string-db.org/] (https://cn.string-db.org/)] and Cytoscape (version v3.9.1) software were employed to study the protein−protein interaction (PPI) network.

### 2.6 Antibodies and flow cytometry

Tissue samples were minced and digested with 1mg/mL Collagenase D to create a cell suspension. Red blood cells were removed using RBC lysis buffer (Biolegend, San Diego, Calif, USA). All cells were stained for viability using a Zombie Aqua fixable viability kit (1:1000; BioLegend) at room temperature for 20 minutes. Staining and antibodies were prepared in PBS. Samples were blocked with anti-mouse CD16/32 blocking antibody (1:50; BioLegend). Cells were stained with the fluorochrome-conjugated mAbs purchased from Biolegend (San Diego, Calif, USA) for CD45 (30-F11), CD45.2 (104), CD3ϵ (500A2,c-kit (2B8), CD3 (17A2), CD49b (HMα2), FcϵRIα (MAR-1), CD11b (M1/70), CD200R3 (Ba13), IL-4 (11b11), GATA3 (16E10A23),TSLPR (22H9) and the fluorochrome-conjugated mAbs purchased from eBioscience (San Diego, Calif, USA) for CD4 (RM4-5), CD5 (53-7.3), CD11c (N418), CD19 (1D3), NK1.1 (PK136), CD170 (1RNM44N). Lung basophils were identified as live, CD45^+^, Lin (CD3, CD5, CD19, CD11c, and NK1.1)^-^, c-kit^-^, CD49b^+^, FcϵRIα^+^/CD200R3^+^ cells. Lung eosinophils were identified as live, CD45^+^, Lin (CD3, CD5, CD19, CD11c, and NK1.1)^-^, CD11b^+^, CD170^+^ cells. Eosinophils in the BALF were identified as CD45^+^, CD11b^+^, and CD170^+^ cells (26) (**Supplementary Figure S1**). For intracellular staining, cells were permeabilized with Cytofix Fixation/Permeabilization Solution Kit (BD, New Jersey, USA) and Foxp3/Transcription Factor Staining Buffer Set (eBioscience). Samples were acquired on a CytFLEX S Flow Cytometer (Beckman, Brea, Calif, USA).

### 2.7 ELISA

Mouse serum was prepared by centrifuging isolated blood at 2,000 rpm for 30 min at 4°C and collecting the supernatants. Serum total IgE, OVA-specific IgE, and TSLP concentrations were quantified using ELISA kits (Invitrogen, USA; Biolegend, USA) according to the manufacturer’s protocol. Color development was measured at 450 nm on the Tecan Sunrise microplate reader (TECAN, Männedorf, Switzerland).

### 2.8 Basophil depletion

For basophil depletion, both anti-FcϵRIα mAb (clone MAR-1, eBioscience) and Anti-mouse CD200R3 antibody (clone BA13, Biolegend) were used. Anti-FcϵRIα mAb treatments in C57BL/6 mice were administered intraperitoneally twice daily for three consecutive days at a dose of 5 μg per mouse before intranasal challenge with OVA. Anti-mouse CD200R3 antibody (1mg/mL) or Rat IgG2a, k isotype control (Biolegend) was administrated in 100uL through i.v. injection on day 11 and day 13.

### 2.9 Statistical analysis

Statistical analysis was performed using GraphPad Prism 10 (GraphPad Software, La Jolla, Calif, USA). FlowJo v10 software (Tree Star) was used for flow cytometry data analysis. Two independent sample *t*-tests were used for comparison between the two groups. One-way analysis of variance was used to compare between groups. P <0.05 was considered statistically significant in all analyses.

## 3 Results

### 3.1 Both AA and AM models could induce eosinophilic lung inflammation in mice

**Figure 1:**
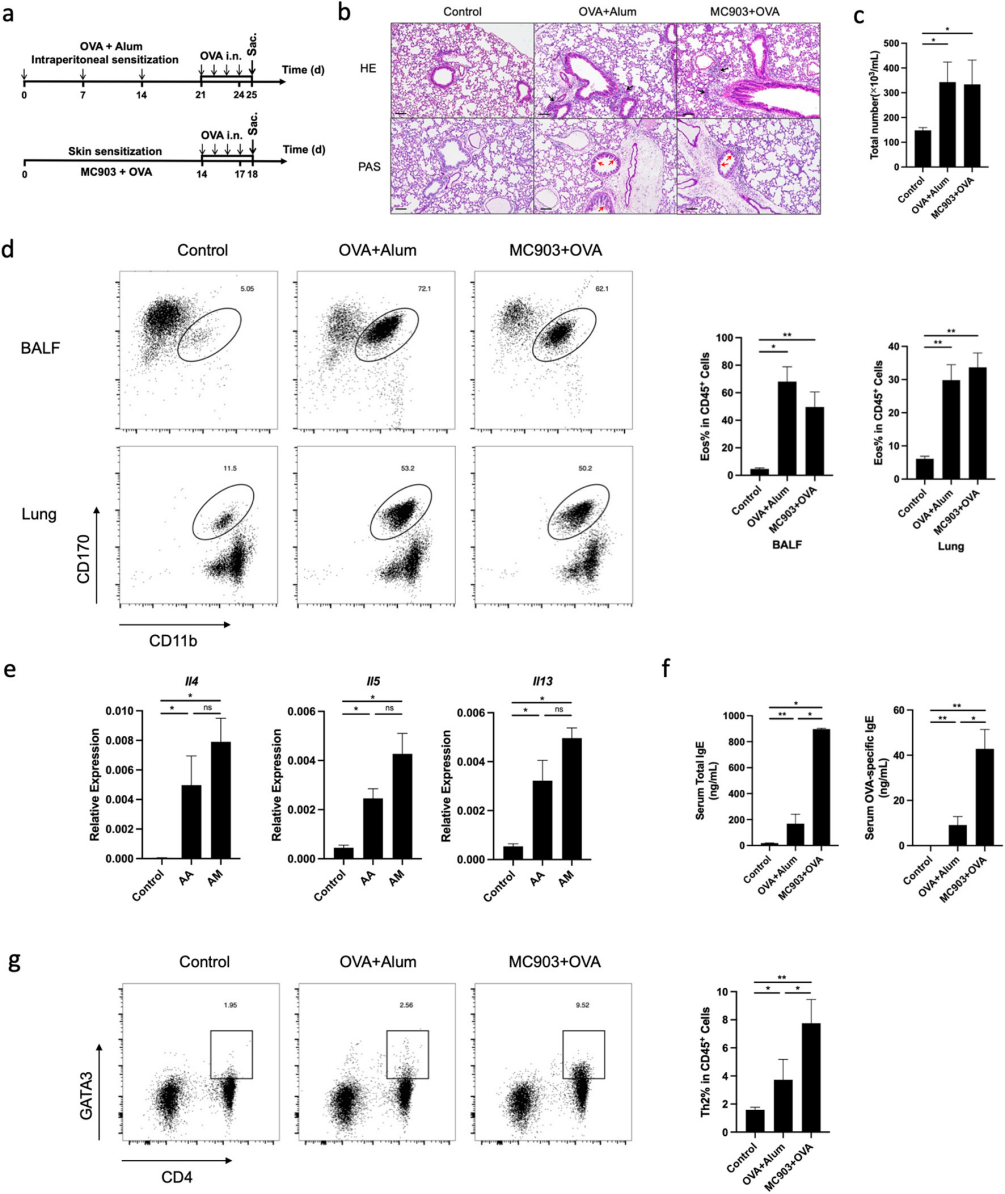
Both AA and AM models could induce eosinophilic lung inflammation in mice. **(a)** Schematic of AA and AM mouse model. **(b)** Histological sections (H&E staining 100× and PAS staining 100×) from the lung. **(c)** Numbers of total cells in the BALF (Wright-Giemsa Staining 100×). **(d)** Representative flow cytometry plots showing frequencies of eosinophils in BALF and lungs and the frequencies of eosinophils in BALF and lungs, as measured by flow cytometry. **(e)** mRNA expression of Th2 cytokines (*Il4, Il5, Il13*). **(f)** Total and allergen-specific serum IgE levels. **(g)** Representative flow cytometry plots displaying the frequencies of Th2 cells in the lungs, measured by flow cytometry. All results shown are mean ± SEM of 5 mice/group and represent three independent experiments. *p <.05, **p <.01, ns, nonsignificant. Scale bar = 100 μm. AA, atopic asthma; AM, atopic march; OVA, ovalbumin.

Wild-type (WT) C57BL/6 mice were sensitized to OVA by combined epicutaneous application of OVA and MC903. Subsequently, lung inflammation was induced by intranasal challenge with OVA. As a control, we used a classical atopic asthma mouse model of eosinophilic pneumonia, in which WT mice were sensitized through intraperitoneal injection of OVA in alum prior to intranasal challenge with OVA (**Figure 1a**). Both AM and AA models were successfully induced experimental eosinophilic lung inflammation, which was characterized by vascular and peribronchial inflammation, eosinophil infiltration, and mucus production histologically (**Figure 1b**). The number of total cells and eosinophils in BALF was significantly increased in OVA-sensitized mice compared to non-sensitized mice after the OVA challenge phase (**Figure 1c**). Flow cytometric analysis revealed an accumulation of eosinophils in the lungs and BALF of OVA-sensitized mice compared to non-sensitized mice upon OVA challenge (**Figure 1d**). The mRNA expression levels of *Il4, Il5*, and *Il13* were higher in the lungs of OVA-sensitized mice than in those of non-sensitized controls after OVA challenge (**Figure 1e**). Significantly higher proportions and numbers of Th2 cells were observed in the lungs of AM mice compared to AA and non-sensitized mice after OVA challenge (**Figure 1g**). In addition, serum total IgE and OVA-specific IgE were higher in OVA-sensitized mice than in control animals (**Figure 1f**). These results suggest that epicutaneous allergen sensitization promotes the development of eosinophilic lung inflammation.

### 3.2 Significant alterations in basophil activation signaling pathway in lung CD45^+^ cells from AM mice

**Figure 2:**
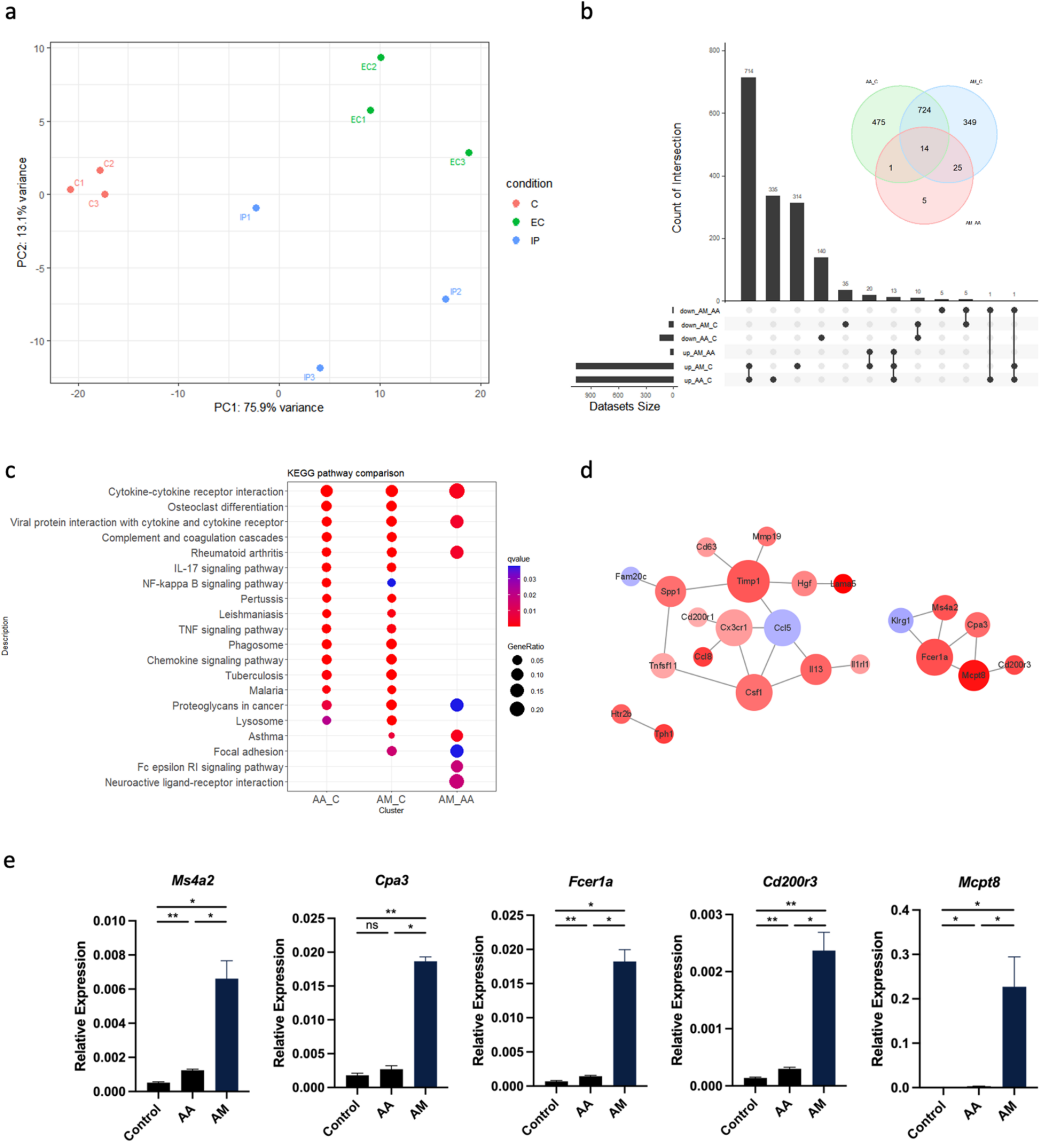
CD45^+^ cells from AA, AM, and controls were isolated from lung tissue by MACS and subjected to RNA-Seq. **(a)** Principal component analysis (PCA) of RNA-seq data from AA, AM, and control (n = 3 per group) shows the primary separation of samples by experimental status. **(b)** Venn plot and upset plot show differentially expressed genes (DEGs) in AA versus controls, AM versus controls, and AM versus AA. **(c)** Kyoto Encyclopedia of Genes and Genomes (KEGG) pathway enrichment analyses of the DEGs with top 20. The coloring of the q-values represents the significance of the matched gene ratio. The value of gene ratio is reflected by the circle size. **(d)** Protein-protein interaction (PPI) network of the DEGs in AM versus AA. Larger node size indicates a higher degree of interaction and the color represents logFC value. **(e)** mRNA expression of *Ms4a2, Cpa3, Fcer1a, Cd200r3*, and *Mcpt8*. All results shown are mean ± SEM of 5 mice/group and represent over two to three independent experiments. *p <.05, **p <.01, ns, nonsignificant. AA, atopic asthma; AM, atopic march; EC, epicutaneous sensitization; IP, intraperitoneal sensitization.

We then performed RNA-Seq of immune cells in mouse lung tissue induced by epicutaneous sensitization and intraperitoneal sensitization. Lung CD45^+^ immune cells were isolated from lung tissue after collagenase digestion and viable cells were sorted by magnetic-activated cell sorting (MACS). The results of RNA-Seq demonstrated a clear distinction in gene expression between the populations of lung immune cells isolated from each group (**Figure 2a**). The first principal component (PC1) accounted for 75.9% of the total variability in the RNA-seq data and also completely separated the treatment group from the controls (**Figure 2a**). A total of 45 genes were differentially expressed between AA and AM models, 1214 between AA and controls, and 1112 between AM and controls (**Figure 2b**). Kyoto Encyclopedia of Genes and Genomes (KEGG) pathway analysis revealed that several immune-related signaling pathways, including cytokine-cytokine receptor interaction, complement and coagulation cascades, IL-17 signaling pathway, and TNF signaling pathway were upregulated in the AA and AM groups compared to the controls group. However, the FcϵRI signaling pathway and neuroactive ligand-receptor interaction pathway were predominantly enriched in the AM group compared to the AA group (**Figure 2c**). A protein-protein interaction (PPI) network was constructed using the STRING online tool with a cutoff score >0.6 to identify the interactions between differentially expressed genes (DEGs) in AM vs AA. The pairing image was generated using Cytoscape (**Figure 2d**). In addition to many important asthma-related genes identified in the current study, basophil-specific gene signatures, such as *Mcpt8, Ms4a2, Cpa3, Fcer1a*, and *Cd200r3* were highly expressed in the AM group (**Figure 2e**). Taken together, these findings suggest that basophils may contribute to lung inflammation induced by OVA epicutaneous sensitization.

### 3.3 Basophil activation is necessary in mice lung inflammation induced by epicutaneous OVA sensitization

**Figure 3:**
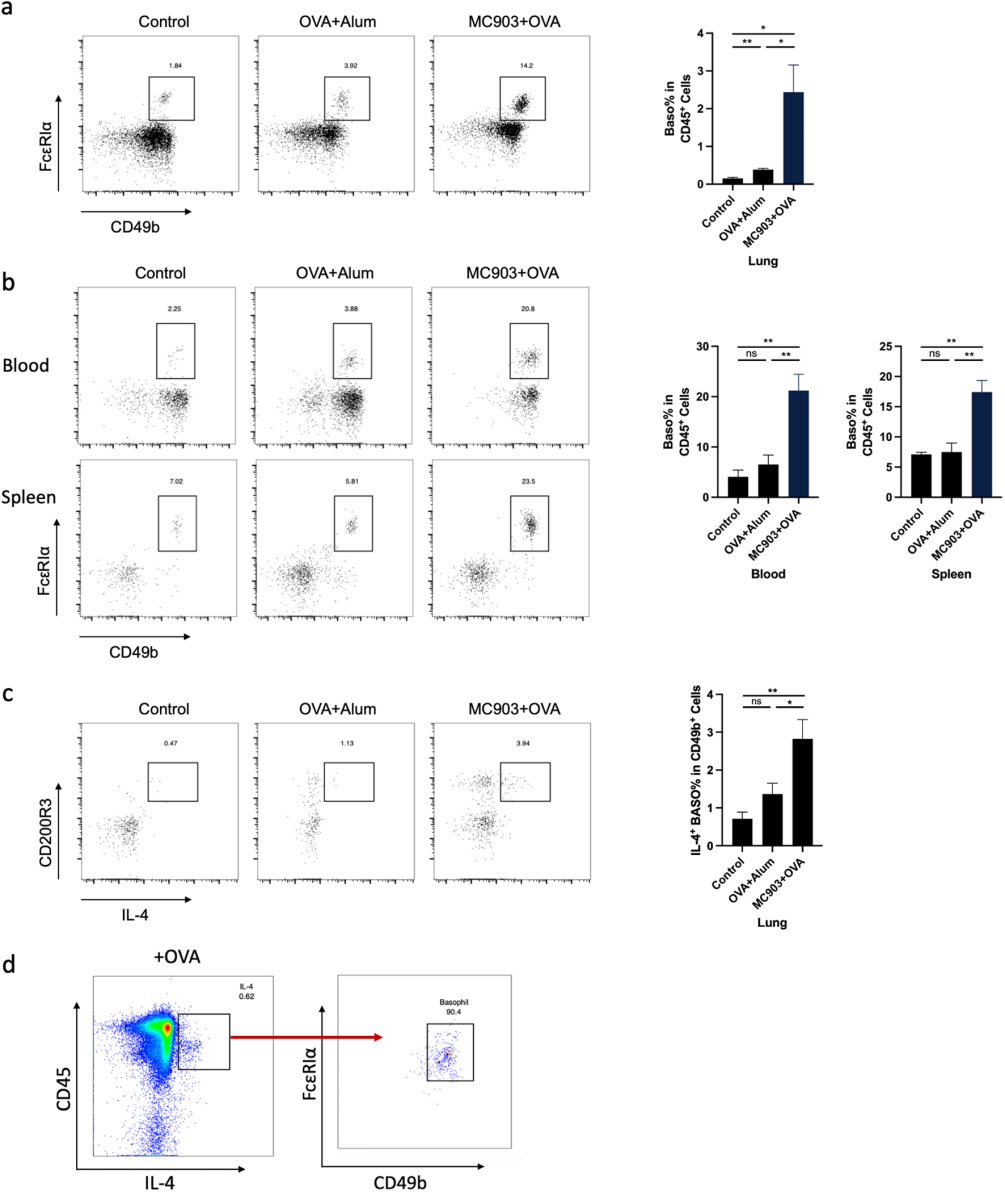
Basophil activation is necessary in mouse lung inflammation induced by epicutaneous OVA sensitization. **(a)** Representative flow cytometry plots illustrating the frequencies of basophils in the lung, measured by flow cytometry. **(b)** Representative flow cytometry plots illustrating the frequencies of basophils in the blood and spleen, as measured by flow cytometry. **(c)** Representative flow cytometry plots illustrating the frequencies of IL-4^+^ basophils in the lung, measured by flow cytometry. **(d)** Flow cytometry measured the frequency of IL-4^+^ basophils in OVA-stimulated lung primary cells. All results shown are mean ± SEM of 6 mice/group and represent over three independent experiments. *p <.05, **p <.01, ns, nonsignificant; OVA, ovalbumin.

Next, we used flow cytometry to verify the number of basophils in the lungs of mice sensitized with OVA epicutaneously or intraperitoneally. The results were consistent with the sequencing results, showing that the count of basophils in the lungs of mice sensitized epicutaneously was significantly higher than that of mice sensitized intraperitoneally or the control group (**Figure 3a**). We also observed a similar tendency of basophil in both spleen and peripheral blood of the mice (**Figure 3b**). To further confirm the role of basophils in the pathogenesis of allergic airway inflammation, we investigated the activation status of basophils. Previous literature has shown that basophils can secrete IL-4 in response to allergic reactions (27). We found that the proportion of IL-4-secreting basophils in the epicutaneous sensitization group was significantly higher than that in the intraperitoneal sensitization group and the control group (**Figure 3c**). In addition, when mouse lung cell suspensions were stimulated with OVA *in vitro*, the IL-4-secreting immune cells were predominantly identified as basophils (**Figure 3d**).

### 3.4 Basophils are required for the development of eosinophilic lung inflammation induced by epicutaneous OVA sensitization

**Figure 4:**
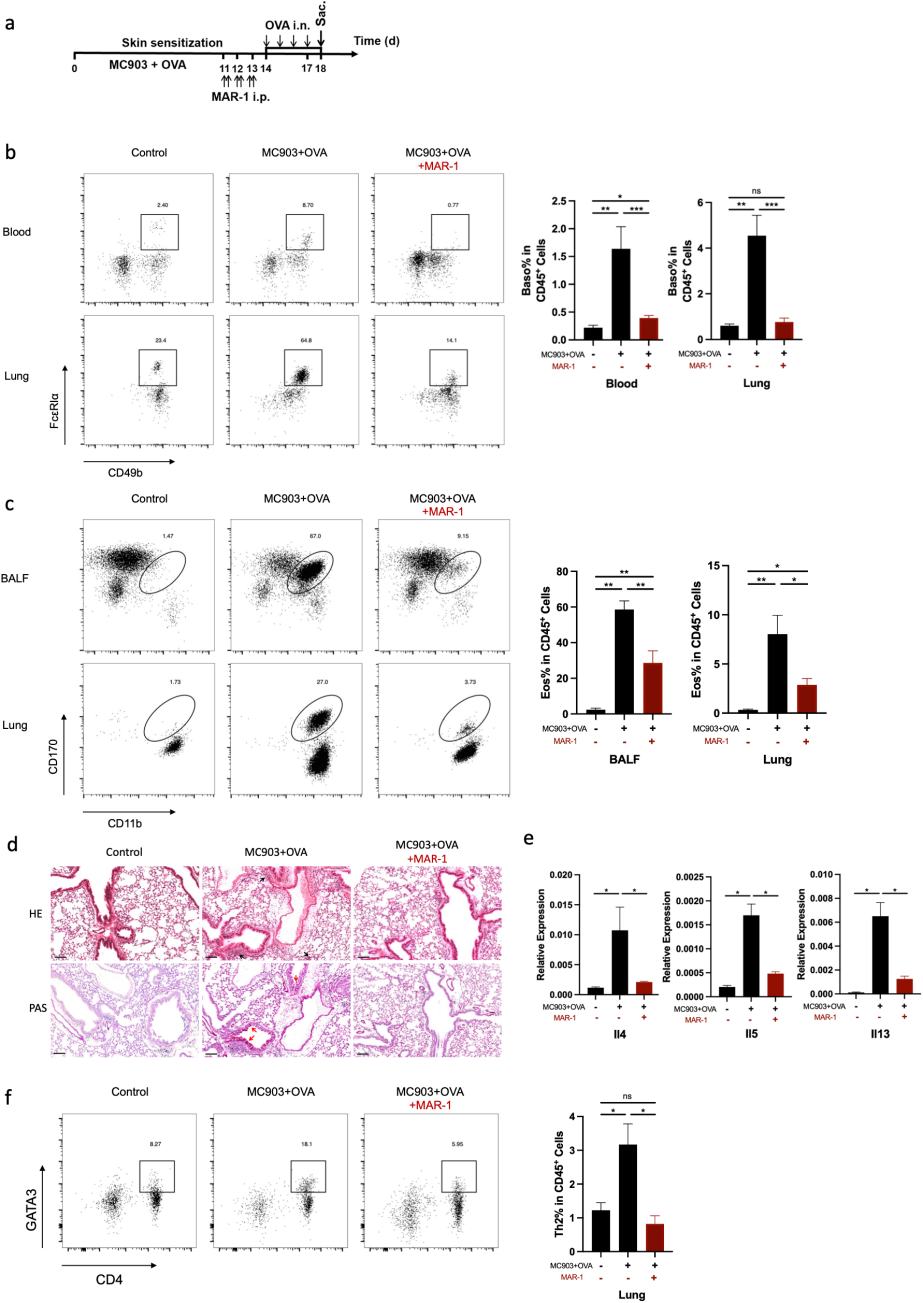
Basophils are required for the development of eosinophilic lung inflammation induced by epicutaneous OVA sensitization. **(a)** Schematic of treatment with anti-FcϵRIα mAb (MAR-1) in C57BL/6 mice exhibiting established epidermal sensitized eosinophilic lung inflammation. **(b)** Representative flow cytometry plots demonstrate the frequencies of basophils in the lung and blood, as measured by flow cytometry. **(c)** Representative flow cytometry plots illustrate the frequencies of eosinophils in BALF and lungs, as determined by flow cytometry. **(d)** Histological sections (H&E staining 20× and PAS staining) from the lung. **(e)** mRNA expression of Th2 cytokines (Il4, Il5, Il13). **(f)** Representative flow cytometry plots showing frequencies of Th2 cells in lungs and the frequencies of Th2 cells in lungs, as measured by flow cytometry. All results shown are mean ± SEM of 7 mice/group and represent two independent experiments. *p <.05, **p <.01, ***p <.001, ns, nonsignificant. Scale bar = 100 μm.

**Figure 5:**
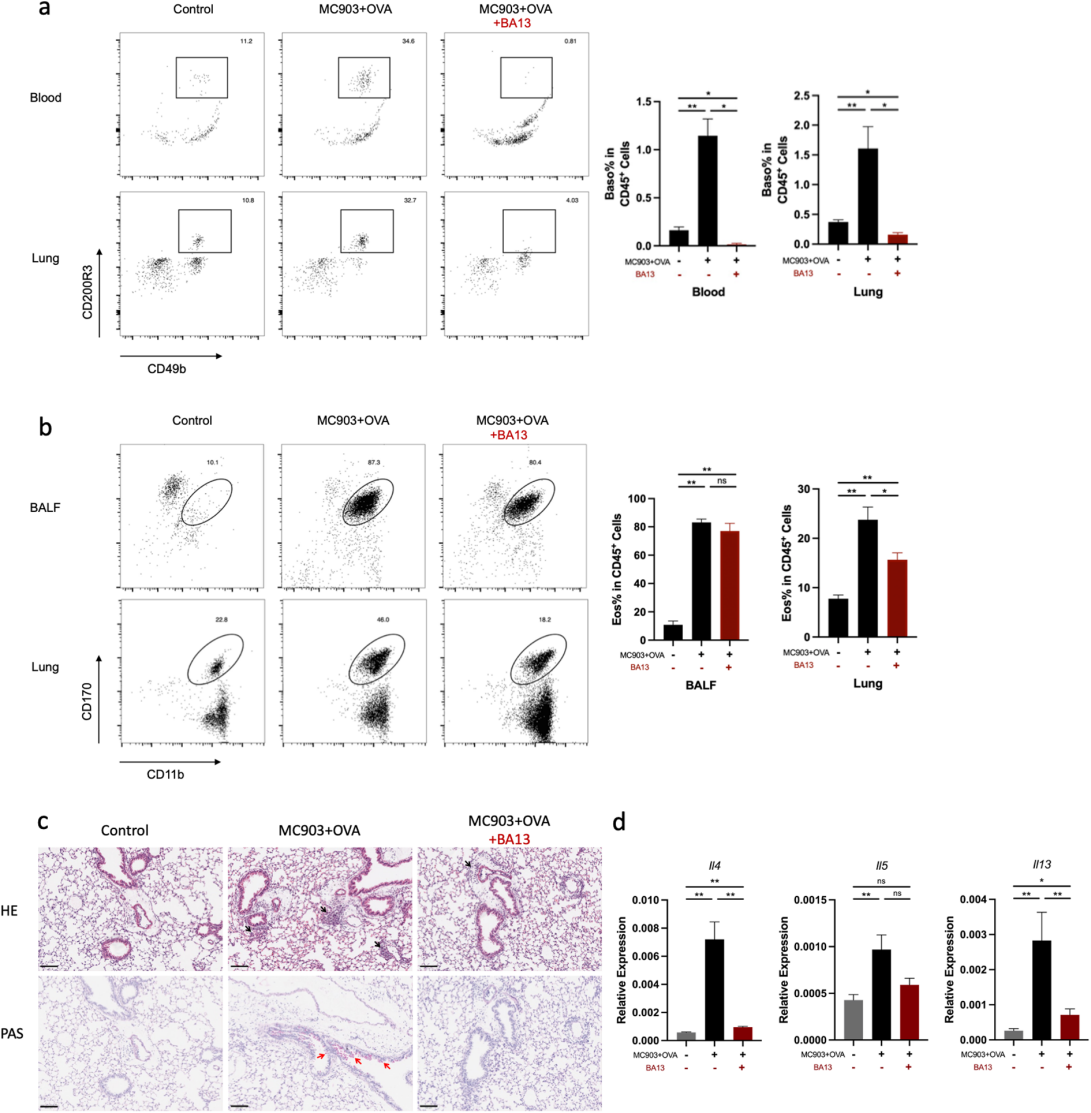
BA-13 depletion of Basophils alleviates eosinophilic lung inflammation induced by epicutaneous OVA sensitization. **(a)** Basophils were significantly reduced in the lung and blood. **(b)** Representative flow cytometry plots showing the frequencies of eosinophils in BALF and lungs, as measured by flow cytometry. **(c)** Histological sections (H&E staining at 100× and PAS staining) from the lung. **(d)** mRNA expression of Th2 cytokines (*Il4, Il5 Il13*). All results shown are mean ± SEM of 5 mice/group and represent two independent experiments. *p <.05, **p <.01, ns, nonsignificant. Scale bar = 100 μm.

To determine the contribution of basophils to the development of eosinophilic lung inflammation, we investigated whether the depletion of basophils attenuated eosinophilic lung inflammation in AM model. MAR-1 antibody is an effective depleting agent with a depletion period of at least 10 days (28). In this study, MAR-1 antibody was administered intraperitoneally twice a day for three consecutive days before intranasal challenge with OVA (**Figures 4a, b**). When basophils were depleted prior to OVA i.n. challenge, a reduction in eosinophil accumulation was observed in both lungs and BALF compared to control mice (**Figure 4c**). The results of HE and PAS staining revealed a significant reduction in inflammatory cell infiltration and mucus secretion after basophil depletion (**Figure 4d**). The mRNA levels of *Il4, Il5*, and *Il13* were also decreased in the basophil-depleted group (**Figure 4e**). Additionally, flow cytometric analysis demonstrated a decrease in Th2 cell frequency in the lungs of mice treated with anti-MAR-1 mAb (**Figure 4f**). These results indicate that basophils are major contributors to the pathogenesis of eosinophilic lung inflammation in mice. To validate the results, we further induced BA-13 antibody to replicate this model, we found that BA-13 was effectively depleted basophils in both blood and lung tissue (**Figure 5a**). A significant reduction of lung eosinophil inflammation was observed, observed, similar to that observed with MAR-1 administration (**Figures 5b–d**).

### 3.5 TSLP is necessary for promoting basophil activation in OVA-induced lung inflammation

**Figure 6:**
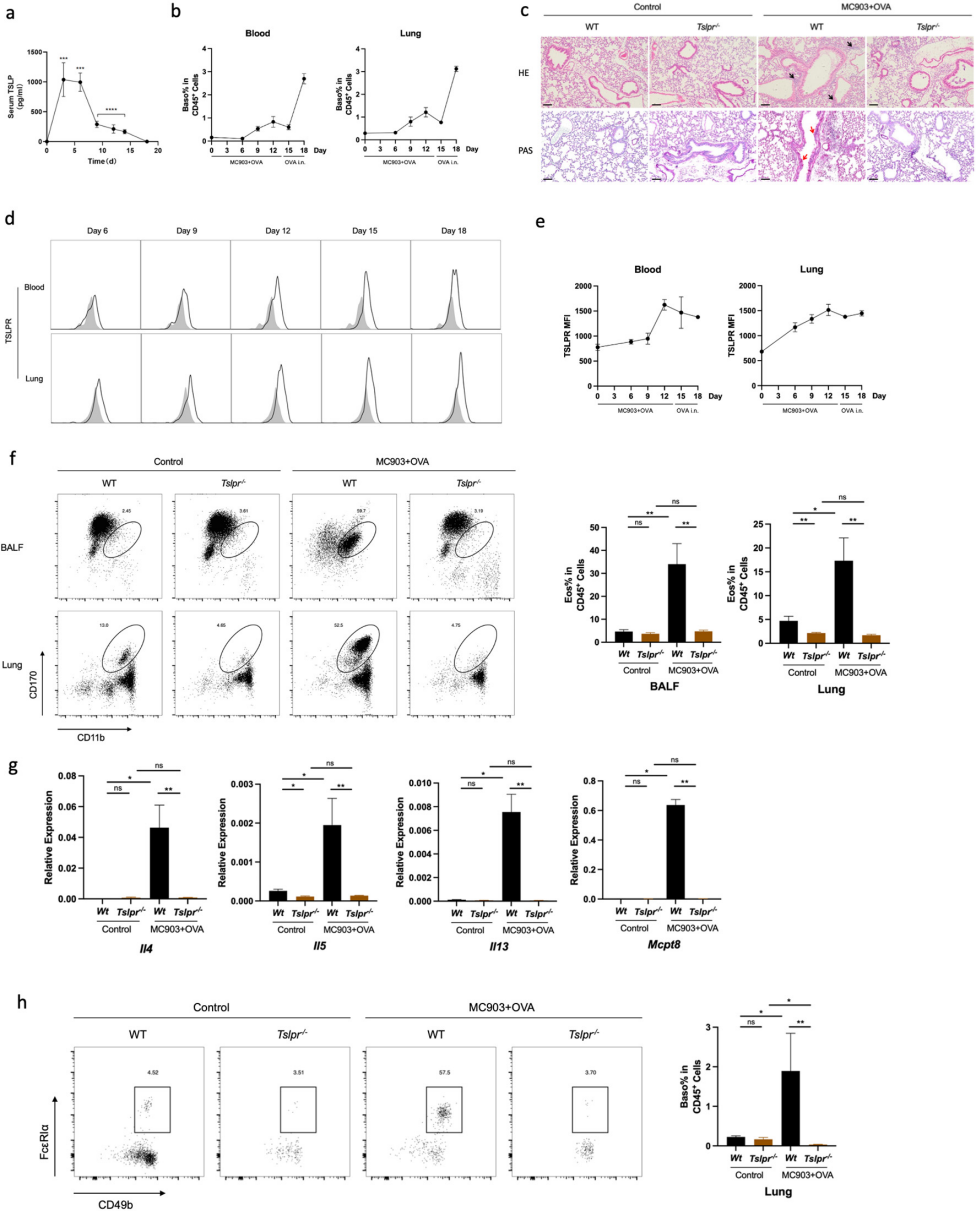
TSLP is necessary for promoting basophil activation in OVA-induced lung inflammation. **(a)** Serum TSLP level in the time manner. **(b)** Blood and lung basophil percentage in CD45^+^ cells. **(c)** Histological sections (H&E staining 10× and PAS staining) from the lung. **(d)** Representative flow cytometry plots show frequencies of blood and lung TSLPR expression. **(e)** Median fluorescence intensity of TSLPR in blood and lungs. **(f)** Representative flow cytometry plots show the frequencies of eosinophils in BALF and lungs and the frequencies of eosinophils in BALF and lungs, as measured by flow cytometry. **(g)** mRNA expression of Th2 cytokines (*Il4, Il5, Il13*) and *Mcpt8*. **(h)** Representative flow cytometry plots show basophil frequencies in lungs and basophils in lungs in *Tslpr* ^-/-^ mouse. All results shown are mean ± SEM of 4 mice/group and represent three independent experiments. *p <.05, **p <.01, ns, nonsignificant. Scale bar = 100 μm.

To clarify how basophil was activated in OVA-induced lung inflammation, we further analyzed the role of TSLP in the mouse model. After topical treatment of the skin with MC903 and OVA, serum TSLP levels noticeably increased from day 3 (**Figure 6a**). Elevated counts of both blood and lung basophils were noted before the intranasal OVA challenge and peaked upon completion of the challenge (**Figure 6b**). Meanwhile, TSLPR expression was upregulated on both blood and lung basophils (**Figure 6d, e**). However, the average fluorescence intensity of FcϵRIα was downregulated after day 9 compared to the control group (**Supplementary Figure S2**). This suggests a potential role for TSLP in activating basophils in the AM mouse model.

Then we established the AM model in the *Tslpr* ^-/-^ mouse strain. HE and PAS staining of the lung tissue illustrated a reduced lung inflammation in *Tslpr* ^-/-^ mice compared to WT (**Figure 6c**). Similarly, the proportion of lung eosinophils was decreased in *Tslpr* ^-/-^ mice (**Figure 6f**), as well as the mRNA expression levels of Th2 cytokines and Mcpt8 (**Figure 6g**). Moreover, the proportion of basophils in the lungs was also decreased in *Tslpr* ^-/-^ mice (**Figure 6h**), and IL-4-secreting basophils were also reduced (**Supplementary Figure S2**). When single-cell suspensions from AM mouse lungs were cultured *in vitro* with TSLP and OVA, higher levels of IL-4 secretion were observed under the stimulation of TSLP and OVA than in control media or OVA alone (**Supplementary Figure S3**), indicating that TSLP may contribute to the promotion of OVA-induced lung inflammation in the AM model.

## 4 Discussion

Children with atopic dermatitis have a 3-fold increased risk of developing asthma (29, 30). However, the mechanism linking atopic dermatitis and asthma is not yet fully understood. In this study, we established an AM mouse model to simulate asthma lung inflammation in the presence of MC903-induced AD-like lesions. Upon comparison with lung inflammation in the non-AD state, it was observed that the primary distinction was an up-regulation of basophil-related genes in the lungs under the AD state. Furthermore, treatment targeting TSLPR or basophils was found to be effective in reducing lung inflammation in mice. It is suggested that TSLP may be involved in the lung inflammatory response induced by OVA through the activation of basophils.

To mimic the pathogenesis features of asthma, mouse models can be divided into a sensitization phase and a challenge phase. Sensitization of allergens by different methods during the sensitization phase can lead to subtle differences in the inflammatory response of the lung. In this study, two sensitization methods were employed: immune adjuvants aluminium hydroxide and OVA via intraperitoneal injection, and MC903 and OVA via epicutaneous sensitization. The AA model is thought to induce an eosinophilic inflammatory infiltrate in the lung by activating CD4^+^ T cells and producing Th2 cytokines such as IL-4, IL-5, and IL-13 (31). Compared to the AA model, our study found that mice co-sensitized with MC903 and OVA had higher levels of total and OVA-specific IgE in the peripheral blood, as well as a greater proportion of Th2 cells in the lungs. Moreover, RNA-seq revealed significant alteration in the expression of gene phenotypes among AM, AA, and control mouse models and indicated that basophil-related genes are differentially expressed in these models. Consistent with our study, topical emollient could alleviate lung inflammation in AM mice, and serum TSLP levels and genes related to pulmonary basophil activation were significantly down-regulated (32).

To further validate the role of basophils in the AM model, we found that the amount of basophils significantly increased in the lungs, peripheral blood, and spleen. Additionally, the proportion of basophils expressing IL-4 was found to be increased. In mouse primary lung cells cultured and stimulated with OVA *in vitro*, basophils were found to be the main cells expressing IL-4. Basophils can produce significant amounts of IL-4 in response to stimuli in both IgE-dependent and IgE-independent manners (33). Basophil-derived IL-4 promotes eosinophil infiltration into inflamed tissue (34) and facilitates the differentiation of naïve T cells to Th2 cells by expressing MHC class II on the cell surface in allergic inflammation models (21, 35). In previous studies, OVA-induced lung inflammation was thought to be mediated by DCs and CD4^+^T cells, independent of basophil during the sensitization phase (36). In this study, we discovered that the knockdown of basophil during the sensitization phase did not attenuate lung inflammation (data not shown), but the knockdown of basophil prior to the challenge phase significantly attenuated lung inflammation. To preliminarily exclude a potential role for mast cells in the basophil-depleted AM model, we examined mast cell protease-1 (MCPT-1) expression in lungs and BALF. We found that it tended to be elevated in the AM model and after basophil depletion (**Supplementary Figure S4**). These findings suggest that basophil plays a role in OVA-induced lung inflammation during the challenge phase.

Basophils have recently been shown to play an important role in allergic diseases and pruritus (26, 37, 38) and participate in the inflammatory response through non-IgE-dependent activation in both EoE and food allergy in mouse models (22, 25). Similarly, the association between AD and the increased risk of asthma persists regardless of elevated IgE levels, indicating that IgE alone cannot fully explain this relationship (39). To investigate basophil activation in this study, we validated this model in *Tslpr* ^-/-^ mice. In the AA model, *Tslpr* ^-/-^ mice were thought to be unable to induce lung inflammation due to the inability to activate CD4^+^ T cells via TSLP (31). By topical MC903, the systemic release of TSLP was induced, thereby increasing serum TSLP levels (40). The surface expression level of TSLPR on basophils increases gradually, while the expression of the IgE receptor FcϵRIα initially increases but then decreases. In ex vivo experiments of lung tissue in AM model mice, basophils were found to be the primary immune cells producing IL-4 in the lungs. Therefore, the addition of TSLP can enhance the effect of OVA in inducing IL-4 production from basophils.

In conclusion, our study has shown that basophils play a crucial role in OVA-induced lung eosinophilic inflammation in the presence of systemic TSLP release caused by topical MC903. In clinical trials, TSLP monoclonal antibodies are effective in controlling eosinophilic inflammation in the lungs and airway hyperresponsiveness (41, 42). Compared to the efficacy in enhancing lung function and controlling asthma in asthma patients (43), TSLP monoclonal antibodies in treating skin lesions in AD are limited (44). Therefore, it is important to understand the role of TSLP in AD and AM processes to more effectively apply individualized therapy in real-world patients. In short, basophils play a crucial role in initiating asthma during the inflammatory state of AD, and TSLP has the potential to drive this process.

## Data Availability

Datasets related to this article can be found at http://dx.doi.org/10.17632/rkk66tnf4w.1, hosted at Mendeley Data (45).
