# Supplementary material for: Thymic stromal lymphopoietin-activated basophil promotes lung inflammation in mouse atopic march model
Source: Front Immunol. 2025 May 15;16:1573130. doi: 10.3389/fimmu.2025.1573130 (PMC12119317; doi:10.3389/fimmu.2025.1573130)
Supplement: Supplementary file 2 [file DataSheet2.pdf]

frontiers

Supplementary Material

Table S1. Primer sequences used for RT-PCR analysis.

Gene		Primer sequence (‘5-3’)
GAPDH	Forward	AGGTCGGTGTGAACGGATTTG
	Reverse	TGTAGACCATGTAGTTGAGGTCA
Il4	Forward	CCACGGATGCGACAAAAATCA
	Reverse	ACTCTCTGTGGTGTTCTTCGTT
Il5	Forward	GCAATGAGACGATGAGGCTTC
	Reverse	GCCCCTGAAAGATTTCTCCAATG
Il13	Forward	AAGGCCCCCACTACGGTCT
	Reverse	ATGCCCAGGGATGGTCTCTC
Mcpt8	Forward	GTGGGAAATCCCAGTGAGAA
	Reverse	TCCGAATCCAAGGCATAAAG
Cpa3	Forward	AATTGCTCCTGTCCACTTTGAC
	Reverse	TCACTAACTCGGAAATCCACAGT
Cd200r3	Forward	TTGTCAGGAGCAAGTGCCACAG
	Reverse	GAATGCTCAGCAAGGAAGGCAG
Ms4a2	Forward	GGAACAATTGTCTGCTCCGTACTC
	Reverse	TGCAGCGATGCTACTGACAATG
Fcer1a	Forward	TGAGTGCCACCGTTCAAGACAG
	Reverse	TGGCATCTGATGTCAAAGGATCC

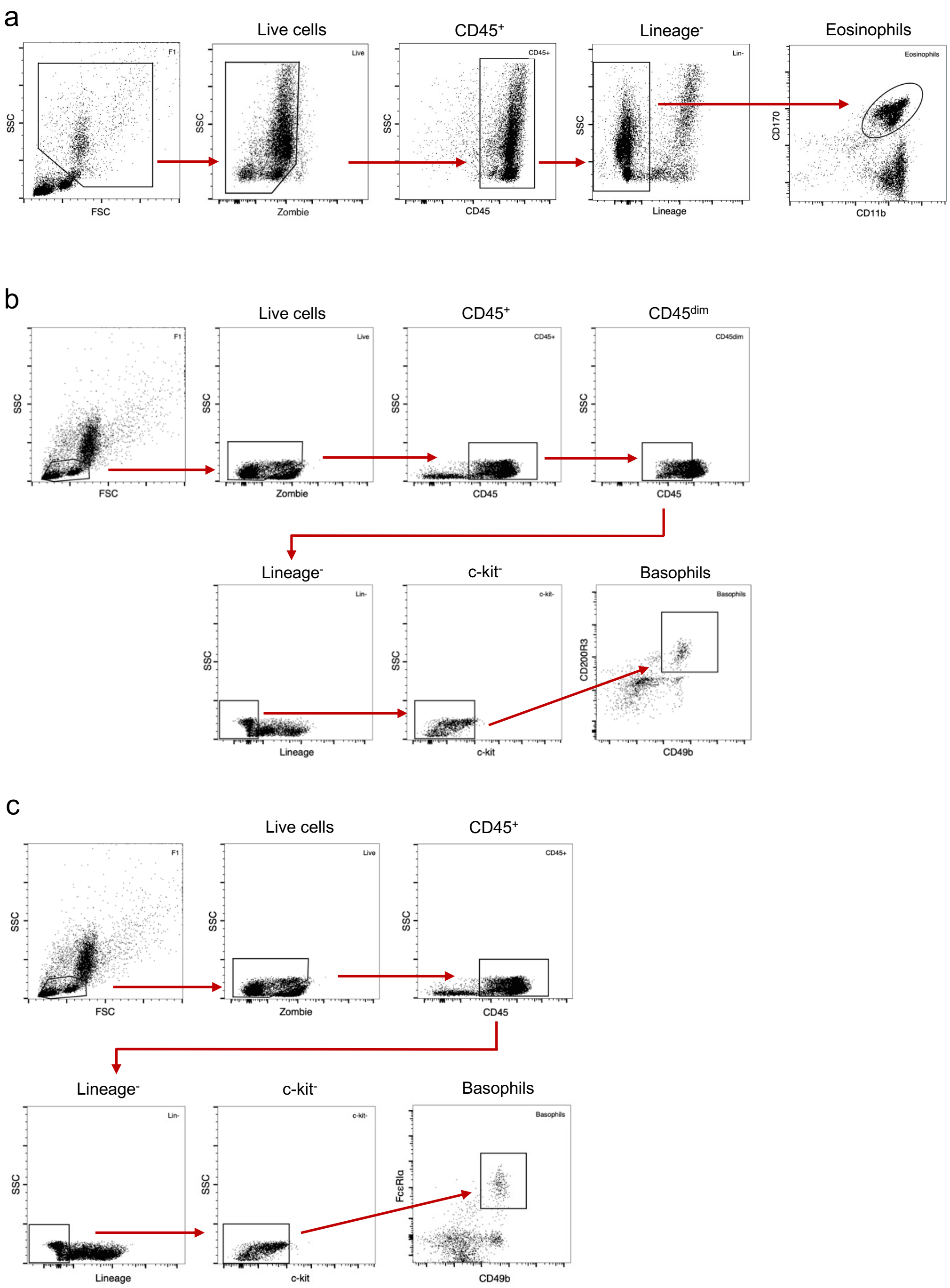

Figure S1. Gating Strategy for Flow Cytometric Analysis. Flow cytometric gating strategy of mouse eosinophils (a). Flow cytometric gating strategy of mouse basophils defined by $CD49b^{+}CD200R3^{+}$ (b) or $CD49b^{+}Fc\varepsilon RI\alpha^{+}$ (c).

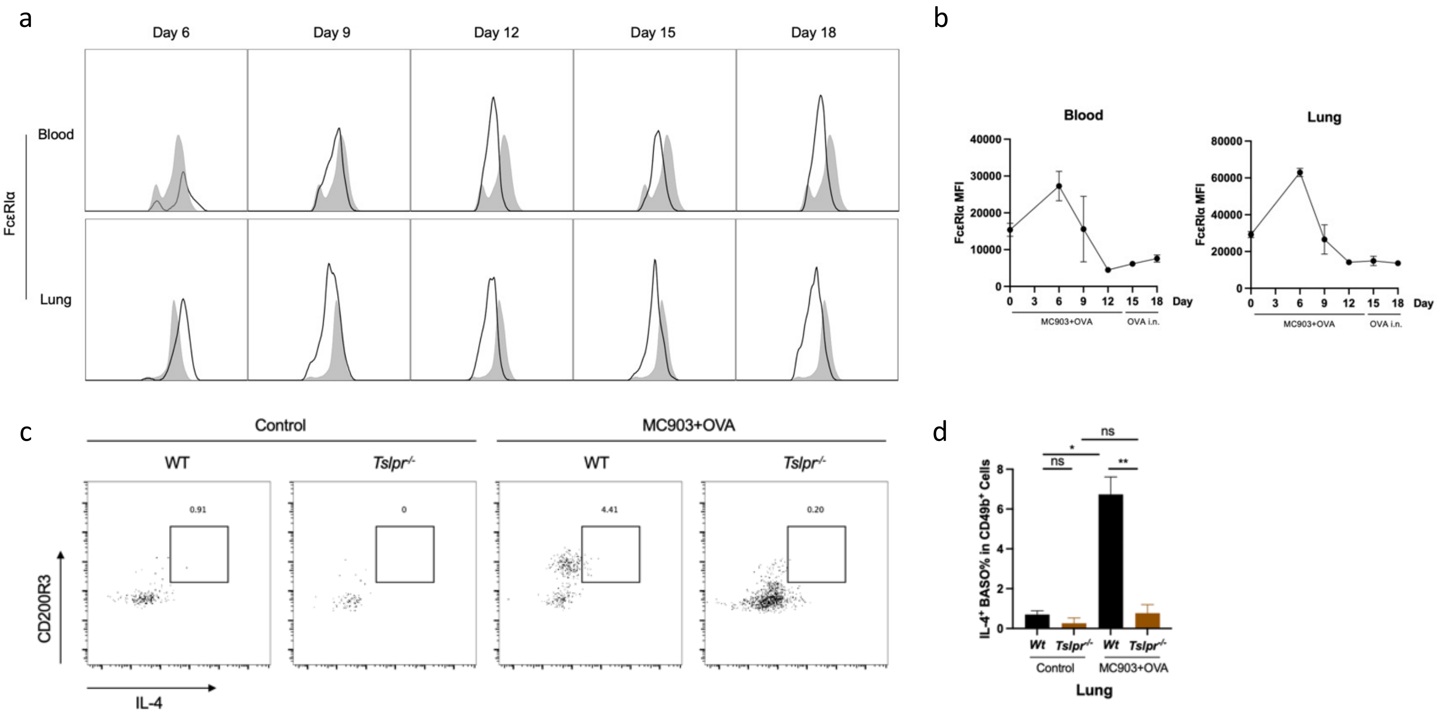

Figure S2. (a) Representative flow cytometry plots showing frequencies showing blood and lung FcεRIα expression. (b) Median fluorescence intensity of FcεRIα in blood and lungs. (c) Representative flow cytometry plots showing frequencies of IL-4-secreting basophils in lungs. (d) Frequencies of IL-4-secreting basophils in lungs in Tslpr$^{-/-}$ mouse. Statistical significance was determined by the Mann-Whitney test. Data represent mean ± SEM. *$p < .05$, **$p < .01$, ns = nonsignificant.

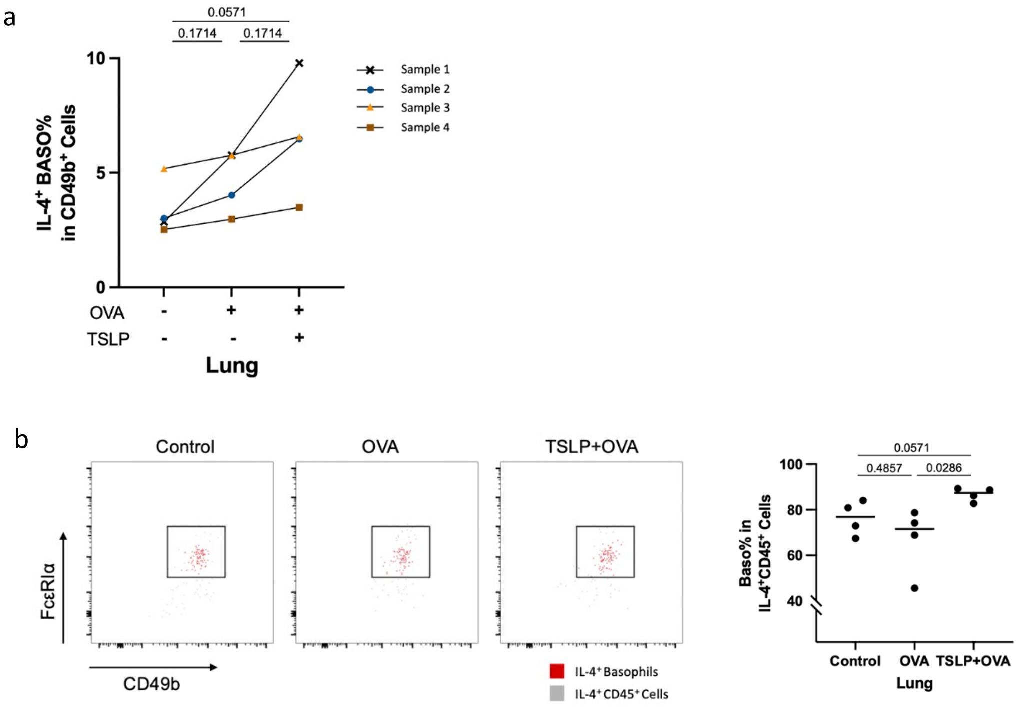

Figure S3. (a) IL-4^{+} basophils in OVA- and TSLP-stimulated lung primary cells. (b) Representative flow cytometry plots IL-4^{+} basophils in OVA and TSLP-stimulated lung primary cells and the percentage of basophil in IL-4^{+}CD45^{+} in OVA and TSLP-stimulated lung primary cells.

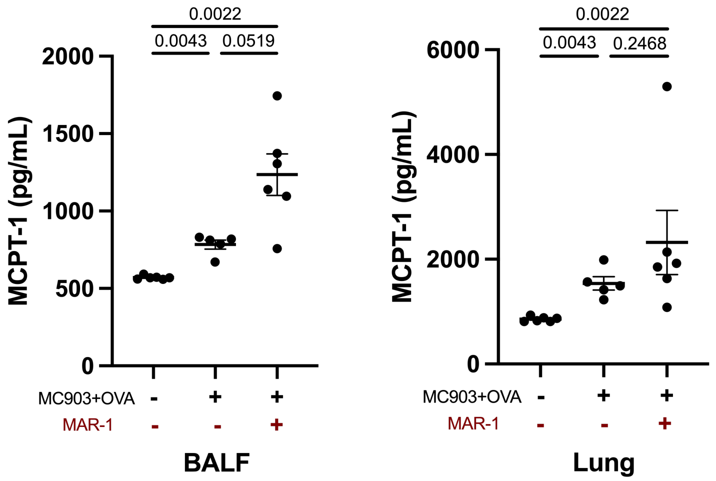

Figure S4. MCPT-1 expression in BALF and lung of AM and MAR-1 injected mice. Statistical significance was determined by Mann-Whitney test. Data represent mean ± SEM.
